# Quality of vision after myopic refractive surgeries: SMILE, FS-LASIK, and ICL

**DOI:** 10.1186/s12886-023-03045-6

**Published:** 2023-06-26

**Authors:** Huiyi Du, Bo Zhang, Zheng Wang, Lu Xiong

**Affiliations:** Department of Refractive Surgery, Guangzhou Aier Eye Hospital, Guangzhou, China

**Keywords:** SMILE, FS-LASIK, ICL, Quality of Vision

## Abstract

**Background:**

To characterize the quality of vision after SMILE, FS-LASIK, and ICL implantation and evaluate the related factors.

**Methods:**

131 eyes of 131 myopic patients (90 female, 41 male) who underwent refractive surgeries including SMILE (35 patients), FS-LASIK (73 patients), and ICL implantation (23 patients) were analyzed. The Quality of Vision questionnaires were completed 3 months after surgery, and the results were characterized and analyzed with baseline characteristics, treatment parameters, and postoperative refractive outcomes using logistic regression analysis to find out predicted factors.

**Results:**

Mean age was 26.5 ± 4.6 years (range: 18 to 39 years) and mean preoperative spherical equivalent was − 4.95 ± 2.04 diopters (D) (range: -1.5 to -13.5). Safety and efficacy index was comparable between different techniques: the safety index was 1.21 ± 0.18, 1.22 ± 0.18, and 1.22 ± 0.16 and the efficacy index were 1.18 ± 0.20, 1.15 ± 0.17, 1.17 ± 0.15 for SMILE, FS-LASIK and ICL respectively. The mean overall QoV score was 13.40 ± 9.11, with mean frequency, severity, and bothersome score of 5.40 ± 3.29, 4.53 ± 3.04, and 3.48 ± 3.18 respectively, and there was no significant difference between different techniques. Overall, the symptom with the highest scores was glare, following fluctuation in vision and halos. Only the scores of halos were significantly different among different techniques (P < 0.000). Using ordinal regression analysis, mesopic pupil size was identified as a risk factor (OR = 1.63, P = 0.037), while postoperative UDVA was a protective factor (OR = 0.036, P = 0.037) for overall QoV scores. Using binary logistic regression analysis, we found that patients with larger mesopic pupil size had an increased risk to experience glare postoperatively; compared to ICL, patients who underwent SMILE or FS-LASIK tended to report fewer halos; patients with better postoperative UDVA were less likely to report blurred vision and focusing difficulty; with larger residual myopic sphere postoperatively, patients experienced focusing difficulties and difficulty judging distance or depth perception more frequently.

**Conclusions:**

SMILE, FS-LASIK, and ICL had comparable visual outcomes. Overall, glare, fluctuation in vision, and halos were the most frequently experienced visual symptoms 3 months postoperatively. Patients with ICL implanted tended to report halos more frequently compared with SMILE and FS-LASIK. Mesopic pupil size, postoperative UDVA, and postoperative residual myopic sphere were predicted factors for reported visual symptoms.

**Supplementary Information:**

The online version contains supplementary material available at 10.1186/s12886-023-03045-6.

## Background

The demand for refractive surgery are increasing yearly and globally during the last decade. Nowadays, the 3 most commonly performed refractive procedures are small incision lenticule extraction (SMILE), femtosecond assisted laser in situ keratomileusis (FS-LASIK), and implantable collamer lens (ICL; STAAR Surgical, Nidau, Switzerland) implantation. The safety, efficacy, and predictability of these procedures have been widely proven and compared [1–4]; however, less has been reported about comparing the quality of vision postoperatively.

The quality of vision has raised attention in refractive surgeries in recent years. Generally, it is divided into objective and subjective quality of vision. The objective quality of vision is usually assessed through higher-order aberrations, modulation transfer function (MTF), objective scatter index (OSI) value and et al. Several studies [5–7] have investigated the objective quality of vision after SMILE, FS-LASIK, and ICL implantation. For high myopic correction, ICL was shown to be superior compared to LASIK and SMILE regarding objective visual outcomes. However, few have studied and compared the subjective quality of vision. As the subjective quality of vision is a subjective perception consisting of both visual and psychological factors, the way to assess it is usually through questionnaires. One instrument, developed by Colm McAlinden [8], called the Quality of Vision (QoV) questionnaire, is a validated and standard measurement for assessing subjective visual symptoms.

The purpose of the current study was to characterize and compare the subjective visual symptoms after different refractive surgeries through the QoV questionnaire and assess the factors that might predict postoperative visual symptoms.

## Methods

### Subjects

After obtaining informed consent, 131 patients who underwent refractive surgeries for the correction of myopia with or without myopic astigmatism from 30th April to 11th December 2022 at Guangzhou Aier Eye Hospital were included. Refractive surgical techniques include SMILE, FS-LASIK, and ICL; the procedure was chosen according to the patient’s preferences after discussion with the surgeons.

The inclusion criterion for corneal laser procedures (SMILE and FS-LASIK) included: no more than 10.00 diopter (D) spherical myopia, no more than 5.00 D astigmatism, manifest refraction stable for the last 2 years (< 0.50 D increase per year of sphere or cylinder), age 18 to 40 years old, IOP ≤ 21; the inclusion criterion for ICL implantation included: age 18 to 40 years old, no more than 18.00 diopter (D) spherical myopia, no more than 5.00 D astigmatism, manifest refraction stable for the last 2 years (< 0.50 D increase per year of sphere or cylinder), IOP ≤ 21, anterior chamber depth > 2.6 mm, endothelial cell density ≥ 2000 cells/mm^2^. The exclusion criteria included severe dry eye, obvious corneal scaring, corneal ectasia, remaining stromal thickness after laser ablation < 250 μm, active ocular or systemic diseases, glaucoma or retina diseases, severe systematic diseases, history of ocular surgery, pregnancy or lactation.

All patients completed the regular 3 months follow-up visits postoperatively, and the QoV questionnaires were assessed at that time. The dominant eye was used for analysis.

#### Surgical techniques

Two surgeons performed all of the procedures (ZW and LX). Each surgeon had a similar experience with the operations. Levofloxacin 5 mg/mL was instilled into the eyes 4 times a day for 3 days before surgery. SMILE was performed on VisuMax femtosecond laser (Carl Zeiss Meditec AG, Jena, Germany). The cap thickness was 110 μm, the cap diameter was 7.5 mm and the incision was set at 135° with a width of 2.00 mm. The optical zone ranged from 6.0 to 6.5 mm. The flaps of FS-LASIK were created either on WaveLight FS200 (Alcon, Fort Worth, TX) or VisuMax femtosecond laser. Flap thickness ranged from 100 to 110 μm with a diameter from 8.1 to 8.5 mm. The flaps were roundly shaped with superior hinges. All the excimer laser treatments of FS-LASIK were done with the WaveLight EX500 (Alcon, Fort Worth, TX). During the ICL implantation procedure, a 2.8 mm temporal clear corneal incision was performed through which a loaded V4c ICL or TICL was injected and positioned to the posterior chamber with the footplates situated posterior to the iris plane. Emmetropia was the goal in all the investigated eyes. After all the procedures, steroids and antibiotics were administered 4 times daily for 7 days, and lubricating eye drops were used for 3 months.

### Preoperative and postoperative parameters

The preoperative and 3-month postoperative routine examinations consisted of measuring uncorrected visual acuity (UCVA), best corrected visual acuity (BCVA) in decimal, manifest refraction, IOP (intraocular pressure), and corneal topography. The IOP was measured with a noncontact tonometer (NCT, Topcon Computerized Tonometer, CT-1). The pupil size was measured under low light conditions (< 5 lx) using the autorefractor (Topcon KR-800). The original Quality of Vision (QoV) Questionnaire was translated into Chinese, and completed by patients 3 months after surgery. The QoV questionnaire [8] was developed by consisting of 10 subjective visual symptoms, including glare, halos, starburst, hazy vision, blurred vision, distortion, double or multiple images, fluctuation in vision, focusing difficulty, and difficulty judging distance or depth perception. The frequency (never [0], occasionally [1], quite often [2], very often [3]), severity(not at all [0], mild [1], moderate [2], severe [3]) and bothersome(not at all [0], a little [1], quite [2], very [3]) of each symptom were asked on a scale of 0 to 3 points.

### Analysis

Pre and postoperative parameters were examined by types of refractive surgeries using an analysis of variance (one-way ANOVA) and the S-N-K test was used for posthoc analysis. A comparison of mean QoV scores among different surgical techniques was conducted using the Kruskal-Wallis test. Chi-squared test was used to compare the frequency, severity, and bothersome rates of visual symptoms for SMILE, FS-LASIK, and ICL. Predictors of overall QoV scores were examined using ordinal logistic regression analysis while a score of 0–9 was defined as mild, 10–19 as moderate, 20–29 as severe, and ≥ 30 as very severe. Logistic regression was used for examining predictors for the occurrence of each visual symptom. Data analysis was performed using SPSS for Windows (Version 22.0, SPSS, Inc.,). A p-value less than 0.05 was considered to be statistically significant.

## Results

### Baseline characteristics and visual outcomes

A total of 131 eyes of 131 patients, 90 females (68.7%) and 41 males (31.3%), age 26.5 ± 4.6 years, were included in this study. 35, 73, and 23 patients underwent SMILE, FS-LASIK, and ICL implantation respectively. The follow-up period was 3 months (± 15days). Baseline parameters were shown in Table 1. One-way ANOVA analysis and posthoc test (S-N-K) revealed that patients who underwent ICL implantation had a significantly higher myopic sphere, thinner central corneal thickness, steeper mean corneal K, larger corneal astigmatism compared with patients who underwent SMILE and FS-LASIK; patients underwent FS-LASIK had higher myopic sphere compared with SMILE. However, the postoperative visual outcomes didn’t differ significantly between different procedures (Table 2). The safety index was 1.21 ± 0.18, 1.22 ± 0.18, and 1.22 ± 0.16 and the efficacy index were 1.18 ± 0.20, 1.15 ± 0.17, 1.17 ± 0.15 for SMILE, FS-LASIK and ICL respectively (All P > 0.05) (Table 2).

**Table 1:** Baseline Parameters

Parameter	Total (131)	SMILE(35)	FS-LASIK (73)	ICL(23)	P
Sex (female)	90 (68.7%)	20(57.1%)	53(72.6%)	17(73.9%)	0.225
Age (years)	26.46 ± 4.64 (18, 39)	25.80 ± 4.98 (18,37)	27.11 ± 4.85 (19, 39)	25.39 ± 2.95 (19, 31)	0.187
Pupil size (mm)	6.21 ± 0.82 (3.75, 7.75)	6.14 ± 0.76 (4.00, 7.25)	6.29 ± 0.86 (3.75, 7.75)	6.05 ± 0.74 (4.50, 7.25)	0.415
Sphere(D)	**-4.95 ± 2.04** **(-13.50, -1.50)**	**-3.82 ± 1.20** **(-6.25, -1.50)**	**-4.67 ± 1.44** **(-8.00, -1.50)**	**-7.54 ± 2.49** **(-13.50, -2.75)**	**0.000**
Cylinder(D)	-0.64 ± 0.55 (-3.50, 0)	-0.54 ± 0.37 (-1.50, 0)	-0. 64 ± 0.54 (-3.50, 0)	-0.78 ± 0.78 (-2.75, 0)	0.241
Pre-op CDVA	1.02 ± 0.08 (0.9, 1.2)	1.03 ± 0.09 (0.9, 1.2)	1.03 ± 0.08 (0.9, 1.2)	1.00 ± 0.06 (0.9, 1.2)	0.139
IOP (mmHg)	17.28 ± 2.08 (12, 21)	17.46 ± 2.11 (14, 21)	17.33 ± 1.95 (13, 21)	16.87 ± 2.46 (12, 21)	0.555
CCT (µm)	**535.41 ± 29.14** **(439, 606)**	**547.71 ± 22.90** **(509, 605)**	**535.62 ± 24.00** **(495, 598)**	**516.04 ± 41.10** **(439, 606)**	**0.000**
Km (D)	**43.43 ± 1.31** **(40.3, 47.0)**	**43.17 ± 1.20** **(40.3, 45.9)**	**43.37 ± 1.36** **(41.0, 47.0)**	**44.05 ± 1.16** **(42.2, 46.2)**	**0.032**
ACA (D)	**1.14 ± 0.54** **(0.1, 3.3)**	**1.07 ± 0.36** **(0.3, 1.9)**	**1.09 ± 0.59** **(0.1, 3.3)**	**1.41 ± 0.51** **(0.7, 2.7)**	**0.026**
Chord µ (mm)	0.18 ± 0.11 (0.01, 0.51)	0.17 ± 0.10 (0.02, 0.43)	0.17 ± 0.11 (0.01, 0.49)	0.23 ± 0.11 (0.08. 0.51)	0.092

The data are presented as means ± standard deviation (SD) and range. Bold data are significant at P < 0.05 (One-way ANOVA and Pearson Chi-Square). D = diopters, Pre-op = preoperative, IOP = intraocular pressure, UDVA = uncorrected distance visual acuity, CDVA = corrected distance visual acuity, Km = mean keratometry, ACA = anterior corneal astigmatism, Chord µ = the two-dimensional distance between the center of the pupil and the subject-fixated coaxially sighted corneal light reflex

**Table 2:** Postoperative Visual Outcomes

Visual outcomes	Total (131)	SMILE(35)	FS-LASIK (73)	ICL(23)	P
Post-op UDVA	1.19 ± 0.17 (0.9, 1.5)	1.21 ± 0.20 (0.9, 1.5)	1.18 ± 0.16 (0.9, 1.5)	1.17 ± 0.14 (0.9, 1.5)	0.601
Post-op CDVA	1.24 ± 0.17 (0.9, 1.5)	1.25 ± 0.18 (0.9, 1.5)	1.25 ± 0.17 (1.0, 1.5)	1.21 ± 0.13 (1.0, 1.5)	0.606
Post-op sphere (D)	-0.006 ± 0.227 (-0.75, + 1.00)	0.029 ± 0.199 (-0.25, + 0.50)	-0.007 ± 0.253 (-0.75, + 1.00)	-0.054 ± 0.168 (-0.50, + 0.25)	0.398
Post-op cylinder (D)	-0.10 ± 0.21 (-1.00, 0)	-0.07 ± 0.18 (-0.75, 0)	-0.11 ± 0.20 (-0.75, 0)	-0.14 ± 0.29 (-1.00. 0)	0.467
Safety index	1.22 ± 0.18	1.21 ± 0.18	1.22 ± 0.18	1.22 ± 0.16	0.996
Efficacy index	1.16 ± 0.17	1.18 ± 0.20	1.15 ± 0.17	1.17 ± 0.15	0.752

The data are presented as means ± standard deviation (SD) and range. Bold data are significant at P < 0.05 (One-way ANOVA and Pearson Chi-Square). Post-op = postoperative, UDVA = uncorrected distance visual acuity, CDVA = corrected distance visual acuity, D = diopters

### Quality of vision questionnaires

**Fig. 1:**
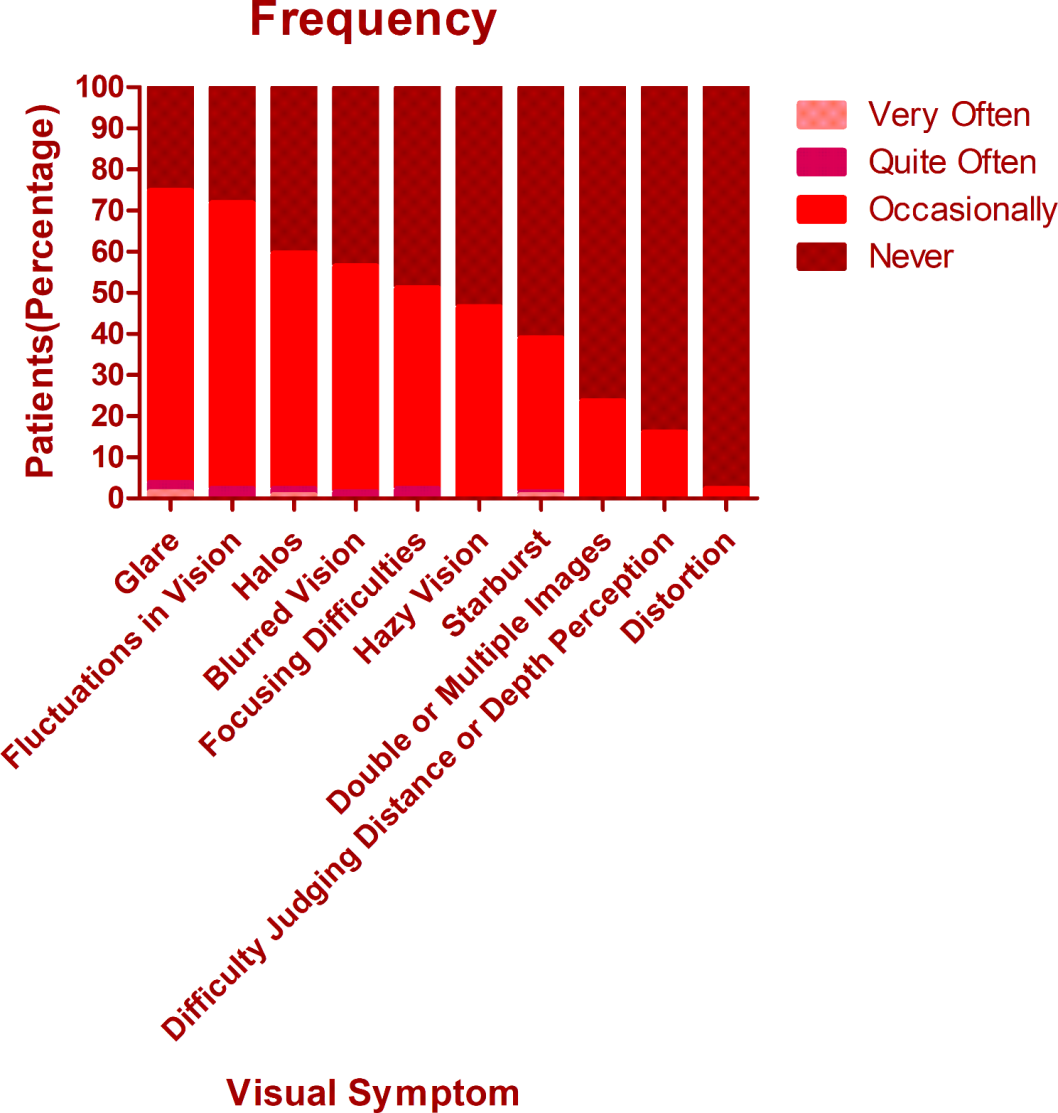
The frequency of different visual symptoms experienced by patients

**Fig. 2:**
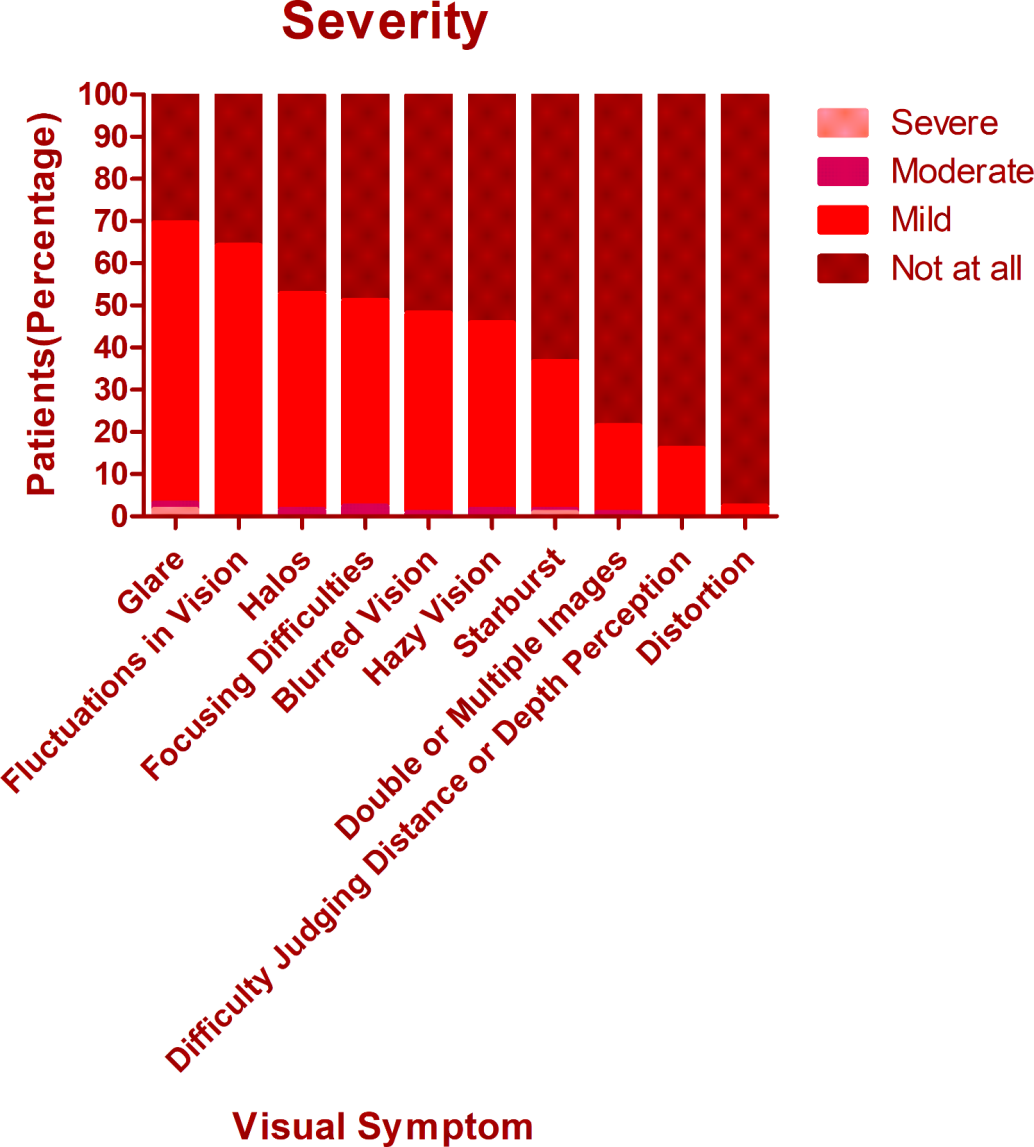
The severity of different visual symptoms experienced by patients

**Fig. 3:**
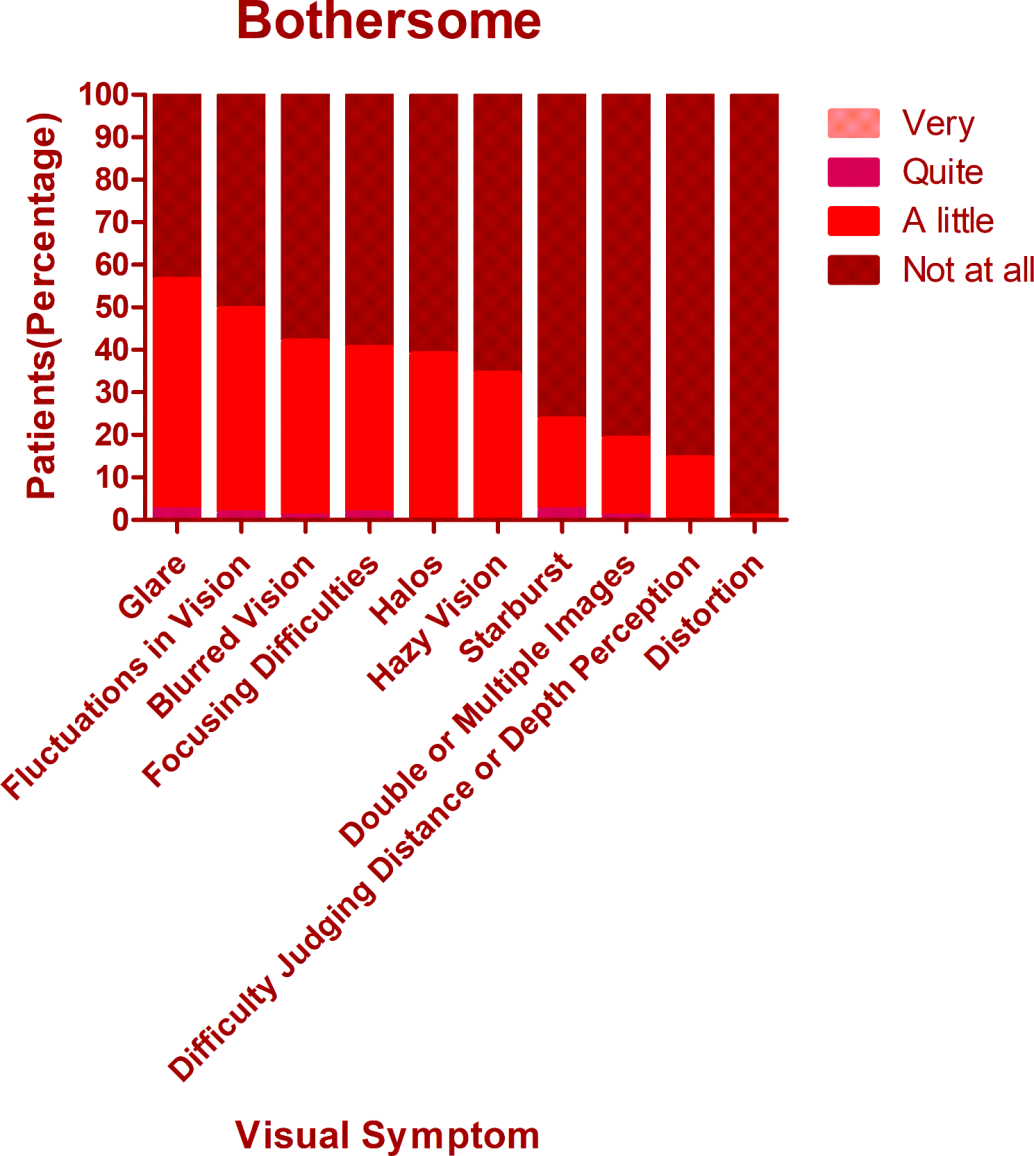
The bothersome of different visual symptoms experienced by patients

**Fig. 4:**
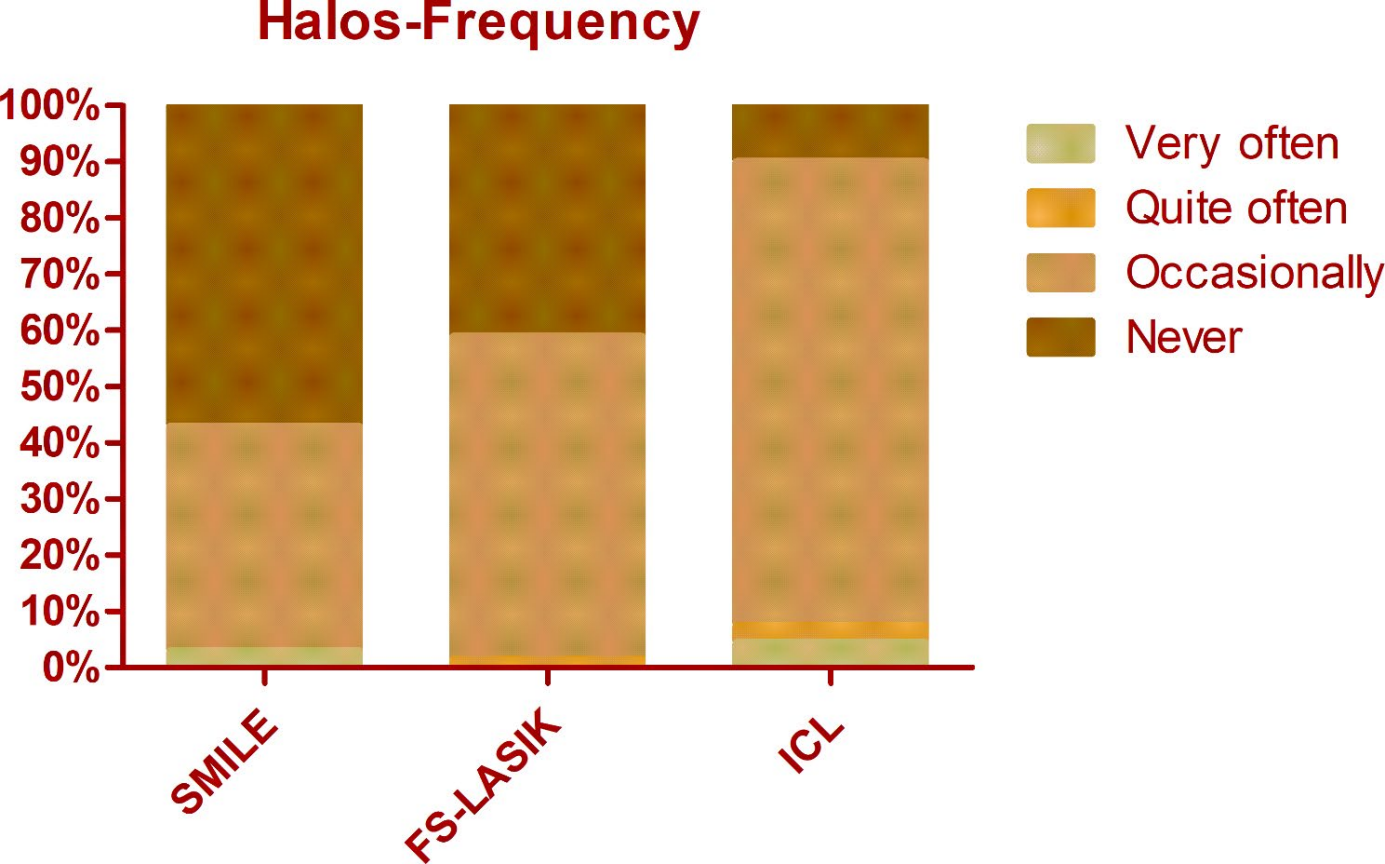
The frequency of halos after SMILE, FS-LASIK, and ICL. Person Chi-squared test, P = 0.016

**Fig. 5:**
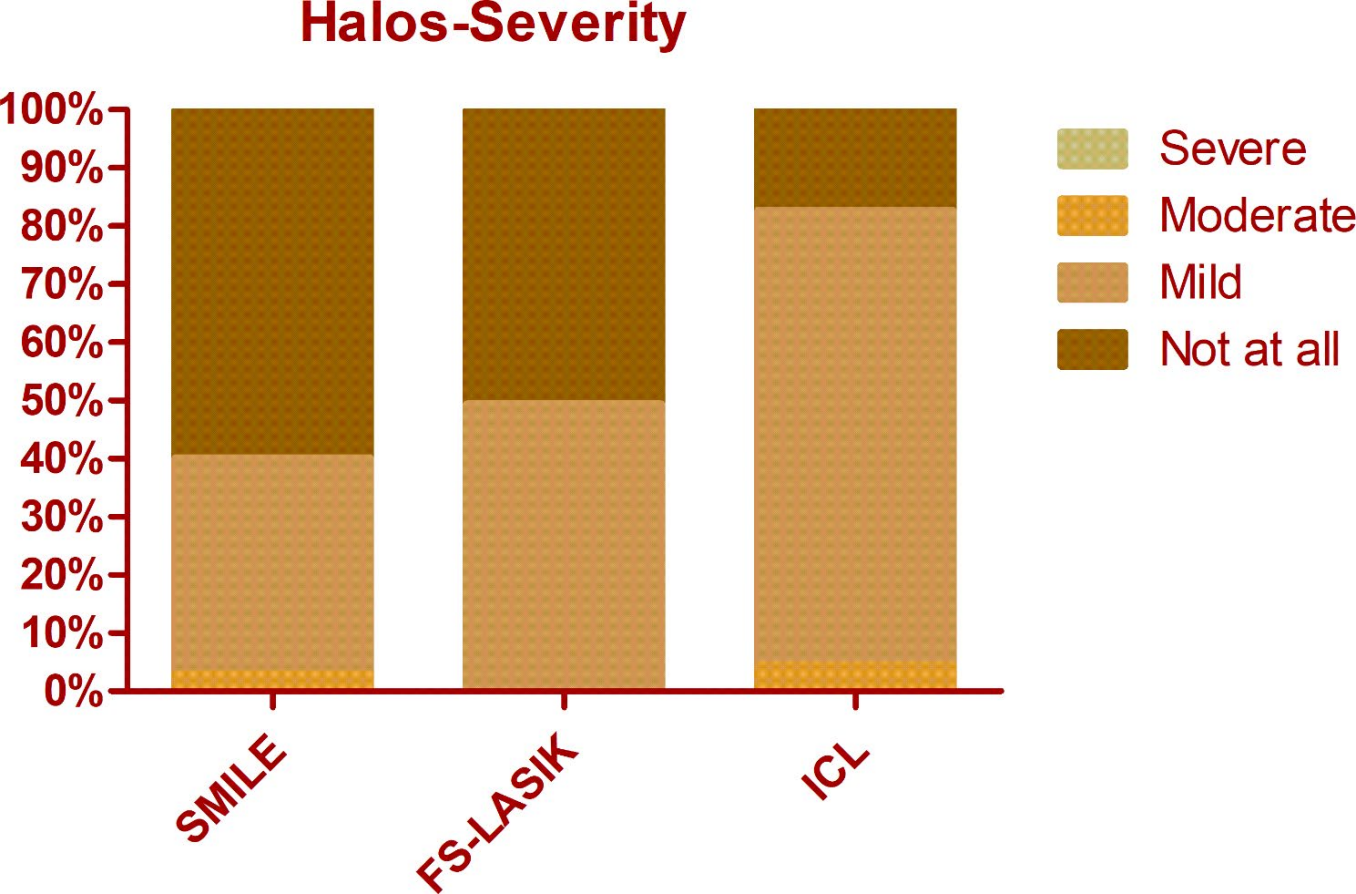
The severity of halos after SMILE, FS-LASIK, and ICL. Person Chi-squared test, P = 0.011

**Fig. 6:**
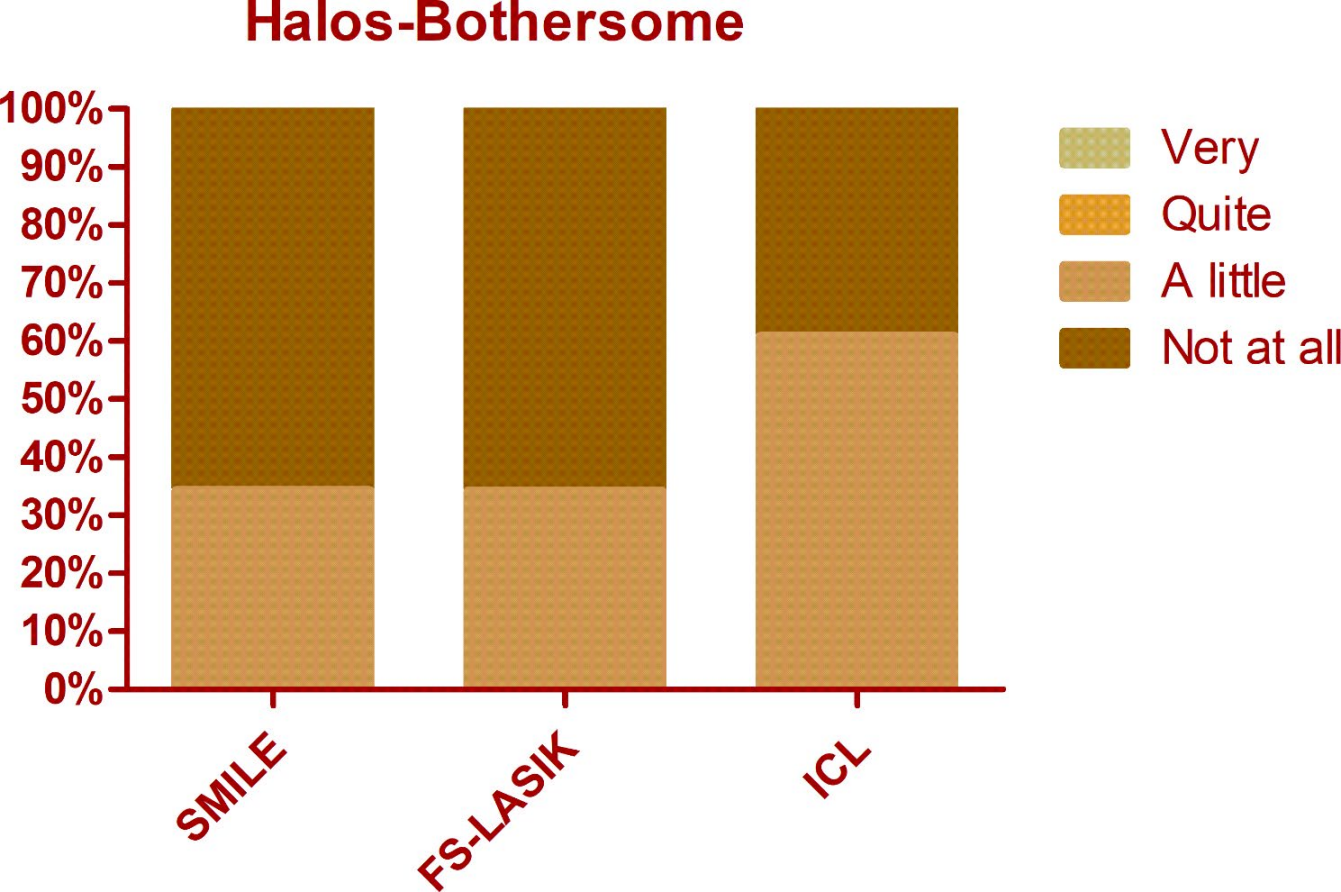
The bothersome of halos after SMILE, FS-LASIK, and ICL. Person Chi-squared test, P = 0.059

The overall mean score of QoV was 13.40 ± 9.11. The mean frequency, severity, and bothersome scores were 5.40 ± 3.29, 4.53 ± 3.04, and 3.48 ± 3.18 respectively. There was no significant difference among different procedures regarding the overall QoV scores as well as the overall frequency, severity, and bothersome scores (Kruskal-Wallis Test, P = 0.714, 0.156, 0.806, 0.428). The glare was the most frequent, bothersome, and severest symptom, which was experienced by 74.8% of patients, 69.5% of patients reported at least mild severity, and 56.5% of patients reported it bothersome (Figs. 1, 2 and 3). Followed by glare, fluctuation in vision and halos were among the top 3 most frequent and severe visual symptoms 3 months postoperatively with a frequency of 71.8% and 59.5%, while 64.1% and 52.7% of patients reported at least mild severity for fluctuation in vision and halos respectively. Regarding different procedures, only the scores of halos were found to be significantly different (Kruskal-Wallis Test, P < 0.000). The frequency of halos after ICL was 87.0%, while that after SMILE and FS-LASIK were 42.9% and 58.9% respectively (Chi-squared test, P = 0.016)(Fig. 4); 82.6% of patients who underwent ICL reported at least mild severity of halos, compared to 40% and 49.3% of patients underwent SMILE and FS-LASIK respectively(P = 0.011), but no one reported severe halos in all procedures(Fig. 5); 60.9%, 34.3% and 34.2% of ICL, SMILE and FS-LASIK patients reported a little bothersome for halos(P = 0.059), while no one reported it quite or very bothersome (Fig. 6).

### Baseline characteristics and QoV

Using ordinal analysis, the mesopic pupil was found to be a risk factor for the overall QoV scores (OR = 1.627, P = 0.037). Patients with a larger mesopic pupil size tended to have more visual symptoms postoperatively. 2 baseline parameters that could be used to predict the occurrence of visual symptoms of glare and halos were identified using logistic regression analysis and were demonstrated in Table 3. The mesopic pupil size was found to be related to the visual symptom of glare. Patients with a larger mesopic pupil size had an increased risk to have glare postoperatively (OR = 1.666, P = 0.038). The other predictor was the procedure used. Compared to ICL, patients who underwent SMILE or FS-LASIK tend to report fewer halos (OR = 0.113, 0.215, P = 0.002, 0.020 respectively).

**Table 3:** Predicted factors of QoV

	Factor	P	Exp(B)	95% C.I for EXP(B)
Total QoV	Mesopic pupil size	0.037	1.627	(1.031, 2.570)
	Post-op UDVA	0.037	0.036	(0.0016,0.816)
Glare	Mesopic pupil size	0.038	1.666	(1.028,2.700)
Halos	SMILE	0.002	0.113	(0.028,0.450)
	FS-LASIK	0.020	0.215	(0.059,0.789)
Blurred Vision	Post-op UDVA	0.024	0.086	(0.010,0.726)
Focusing difficulties	Post-op UDVA	0.011	0.058	(0.006,0.553)
	Post-op sphere (-)	0.012	1.277	(1.055,1.545)
Difficulty judging distance or depth perception	Post-op sphere (-)	0.008	1.549	(1.119,2.145)

QoV = Quality of Vision, UDVA = uncorrected distance visual acuity, Post-op = postoperative

### Visual outcomes and QoV

Using ordinal regression analysis, the postoperative UDVA was found to be a protective factor for the overall QoV scores (OR = 0.037, P = 0.031) (Table 3). 2 visual symptoms --- blurred vision and focusing difficulties were found to be related to postoperative UDVA as well (Table 3). Patients with better UDVA postoperatively tended to report fewer blurred vision (OR = 0.086, P = 0.024) and fewer focusing difficulties (OR = 0.058 P = 0.011). The postoperative myopic sphere was found to be a risk factor for both focusing difficulties (OR = 1.277, P = 0.012) and difficulty judging distance or depth perception (OR = 1.549, P = 0.008) --- with larger residual myopic sphere, patients were more likely to experience the 2 visual symptoms.

## Discussion

Several instruments have been developed to assess patient-report outcomes after refractive surgery. The most frequently used validated questionnaires include Quality of Life Impact of Refractive Correction(QIRC), Quality of Vision(QoV), and Patient-Reported Outcomes with LASIK(PROWL), among which the QoV questionnaire was found to be the most appropriate questionnaire for assessing visual symptoms [9]. The QoV questionnaire [8] is based on item response theory(IRT) using Rasch analysis and consists of 30 items measuring 10 visual symptoms, each on three scales in terms of symptom frequency, severity, and bothersome.

The report of symptoms related to visual quality was thought to be increased after corneal refractive surgeries and after ICL implantation. Steven C et al [10] found an increased report of glare, haze, and halos at night during the first and third month after LASIK, and decreased to preoperative level by 6 months. Reinstein DZ et al. [11] found an increase in QoV symptoms, mainly glare and starbursts after SMILE. In theory, ICL implantation might have a better quality of vision compared with LASIK or SMILE at an early postoperative time point, as it has less disturbance on the cornea and there was some evidence proving this [1].

In the present study, we chose the 3 months postoperatively time point for evaluation and found out that glare, fluctuation in vision, and halos were the most frequently reported visual symptoms. For the 10 visual symptoms evaluated, only halos were found to be significantly different among different procedures --- patients who underwent ICL reported more halos compared to SMILE or FS-LASIK. This is consistent with previous reports as halos were reported to be a major visual complaint following ICL implantation in several studies. Our result is consistent with Ruouyan Wei et al. [7] and Aruma A et al [6], both reporting a significantly higher incidence of postoperative halos after ICL compared with SMILE. One meta-analysis conducted by Kai C et al [4] also showed that ICL implantation had a higher risk of halos compared with SMILE (RR = 1.79, 95%: 1.48 to 2.16). Nevertheless, less was investigated comparing QoV after ICL and FS-LASIK. One case report by Nikolaos ST et al [12] illustrated the patients had one eye underwent LASIK and the fellow eye had phakic intraocular lens implantation reporting fewer night vision problems including glare and halos in the eye with the ICL compared to the LASIK eye, which was contrary to our results. However, it was one case report and the follow-up time was 9 years postoperatively, which is much longer than ours. Although most of the studies showed a high frequency of halos after ICL, it didn’t seem to affect patients’ satisfaction and daily activities [13]. Similarly, in our study, we showed that although the frequency and severity of halos after ICL implantation were significantly higher than that after SMILE and FS-LASIK, the bothersome of halos didn’t differ significantly.

As for the comparison of SMILE and FS-LASIK, one contralateral eye study by He SY et al. reported no significant difference was found in QoV scores between the two, which is consistent with our results [14]. Via other instruments such as QIRC and PROWL [15–17], most studies showed a comparable visual quality between SMILE and LASIK at different time points; only Tian H et al’s study reported that patients after FS-LASIK had more glare than SMILE 3 years postoperatively via QIRC questionnaire [18].

In the present study, we demonstrated mean QoV scores of 13.00 ± 9.02, with overall mean frequency, severity, and bothersome scores of 5.26 ± 3.27, 4.39 ± 3.02, 3.35 ± 3.12 respectively. Previous studies reported much higher scores. In the study of Mohr [19] which evaluated the postoperative QoV after ICL, they demonstrated mean QoV scores of 35.5 ± 11.3. Reinstein DZ et al [11] investing the subjective and objective quality of vision 12 months after SMILE, the mean QoV score was 41 ± 18 and the main visual symptoms were glare and starbursts; however, the population in their study were high myopic patients between − 9.00 and − 13.00 diopters, which were different from ours. Schmelter V et al’ study [20] also demonstrated a higher QoV score for symptom frequency, severity, and bothersome compared with ours after the SMILE procedure (34.63 ± 13.69, 29.60 ± 12.38, and 24.56 ± 16.00, respectively), and found that patients older than 40 years reported worse QoV scores. The difference in the mean QoV scores between ours and the previous studies might due to differences in laser equipment used, surgeons’ techniques, patients’ expectation management, culture, environment, and different populations in the studies.

The predictors for visual symptoms were investigated in previous studies. Theoretically, pupil size is an important predictor for the quality of vision after refractive surgery as suggested by optical models such as the point spread function. The relationship between night vision problems and mesopic pupil size has been investigated in many studies. However, the results were controversial: Haw and Manche [21] reported no relationship between pupil size and postoperative visual symptoms in patients who underwent PRK; other studies [22, 23] also found no significant correlation between pupil size and postoperative visual symptoms and patient satisfaction; while Schallhorn et al [10] found that large pupils had more symptoms in the early postoperative period after LASIK. In our study, the preoperative pupil size was found to be a risk factor for the overall QoV scores (OR = 1.627) and the visual symptom of glare (OR = 1.666) 3 months postoperatively; however, other visual symptoms such as halos and starburst were not related to mesopic pupil size. Therefore, the mesopic pupil size might not be as important a factor in postoperative visual symptoms as we use to think.

Meanwhile, objective quality of vision, such as ocular or corneal higher-order aberrations (HOAs), are also thought to be related to subjective QoV. However, Jakob S et al’s study [24] demonstrated no correlation between postoperative HOA and QoV scores after SMILE; Gyldenkerne A et al [25] also showed that scatter and corneal HOAs were not correlated with visual symptoms.

Haw and Schallhorn’s studies [10, 21] reported the attempted correction was related to visual symptoms after refractive surgery. However, in the present study, we haven’t found a correlation between the preoperative spherical equivalent and the investigated visual symptoms.

Previous studies [22, 23] have shown that postoperative residual refractive error and uncorrected visual acuity was important factor in visual disturbance and patient dissatisfaction. In our study, we found that postoperative UVDA was a protective factor for the overall QoV scores and also related to the visual symptoms of blurred vision and focusing difficulties; postoperative residual myopic sphere was found to be a risk factor for focusing difficulties and difficulty judging distance and depth perception. That is to say, by improving the precision and efficacy of refractive surgeries, postoperative visual complaints could be reduced.

Overall, our studies demonstrated that the visual symptoms after SMILE, FS-LASIK, and ICL implantation 3 months postoperatively were relatively mild and comparable except for halos. To our knowledge, our study is the first to characterize and compare the QoV after SMILE, FS-LASIK, and ICL 3 months postoperatively.

Our studies have several limitations. First, it was a retrospective study. Since the selection of procedures for patients was partly based on the patient’s baseline parameters, some of the parameters were not comparable among the 3 procedures. However, we used regression analysis to control variables. Second, the objective quality of vision was not assessed and analyzed in this study. Third, the QoV questionnaire was assessed only at 3 months postoperatively. Therefore, we did not report on changes in the QoV scores pre- and postoperatively over time. Lastly, although the QoV questionnaire used in this study was a standardized and evaluated one, and was widely used; the Chinese version of it had not been evaluated yet. However, since the questions in it were relatively easy with illustrated pictures on it, we didn’t find patients having any difficulties answering them. In the future, a randomized controlled study including a more objective quality of vision analysis is expected to provide more evidence, and a validated Chinese version of the Quality of Vision questionnaire could be developed. Furthermore, more parameters like pre- and postoperative tear breakup time (TBUT) and et al. could be investigated for their relationships with QoV.

## Conclusion

SMILE, FS-LASIK, and ICL had comparable visual outcomes and quality of vision except for the visual symptom of halos. Overall, glare, fluctuation in vision, and halos were the most frequently experienced visual symptoms 3 months postoperatively. Patients with ICL implanted reported halos more frequently compared with SMILE and FS-LASIK. Mesopic pupil size and residual myopic sphere were found to be risk factors, while postoperative UDVA was a protective factor for the investigated visual symptoms. Through improved efficacy and precision of refractive surgeries, the subjective quality of vision could be improved as well.

## Electronic supplementary material

Below is the link to the electronic supplementary material.

Supplementary Material 1

## Data Availability

All data generated or analyzed during the current study are included in this published article (supplementary file).

## Abbreviations

BCVA: Best corrected visual acuity
FS-LASIK: Femtosecond assisted laser in situ keratomileusis
ICL: Implantable collamer lens
IOP: Intraocular pressure
MTF: Modulation transfer function
NCT: Noncontact tonometer
OSI: Objective scatter index
Pre-op: Preoperative
Post-op: Postoperative
QoV: Quality of vision
SMILE: Small incision lenticule extraction
UCVA: Uncorrected visual acuity
