# Supplementary material for: Quality of vision after myopic refractive surgeries: SMILE, FS-LASIK, and ICL
Source: BMC Ophthalmol. 2023 Jun 26;23:291. doi: 10.1186/s12886-023-03045-6 (PMC10294434; doi:10.1186/s12886-023-03045-6)
Supplement: Supplementary file 1 — Supplementary Material 1 [file 12886_2023_3045_MOESM1_ESM.pdf]

NO.	sex (0=f, 1=m)	age (year)	pupil size (mm)	sphere (D)	cylinder (D)	BCVA (preop)	IOP (mmHg)	CCT (μm)
1	0	28	6	-2.5	-0.5	1.0	17	531
2	1	19	6.25	-1.5	-0.25	1.0	18	539
3	1	26	5.5	-2.25	-0.5	1.0	16	564
4	0	34	6.5	-5.5	-0.5	1.0	15	515
5	0	29	5.5	-3.75	0	1.0	12	481
6	0	23	7.25	-6.5	-0.75	1.0	19	550
7	1	30	5.5	-3.75	-0.5	1.0	19	511
8	0	35	5.75	-4.25	-0.5	1.0	16	549
9	0	25	6.75	-4.75	-1	1.0	16	477
10	1	33	6.5	-5	-0.25	1.0	17	508
11	0	23	5.5	-7.75	-1	1.0	20	510
12	0	27	6.25	-4.75	-0.5	1.0	17	568
13	0	25	7	-7.5	-1.75	1.0	19	564
14	0	25	7	-5.25	-0.5	1.0	20	554
15	1	27	4.75	-2.75	-0.75	1.2	14	519
16	0	30	5.5	-6.5	-1.25	1.0	21	587
17	0	26	5.25	-8.5	0	1.0	19	531
18	0	24	6.5	-7.75	-0.25	1.0	18	532
19	0	37	4.25	-8	-0.75	1.0	16	516
20	0	33	5.5	-5.25	-0.5	1.0	21	540
21	1	30	6.75	-8.25	-0.75	1.0	20	509
22	1	34	4.5	-4	0	1.0	18	552
23	1	24	7	-6	0	1.0	19	570
24	0	26	5.5	-5.5	-0.5	1.0	15	524
25	0	24	6.25	-10.5	0	0.9	17	508
26	0	20	6.75	-4.25	-0.5	1.0	16	515
27	1	18	6.5	-2.75	-0.5	1.0	15	534
28	0	37	5.25	-2.75	-1	1.0	18	537
29	0	36	6	-4.75	-1.25	1.0	18	519
30	0	32	6.75	-6.25	-1	1.2	18	510
31	1	18	7.25	-6.25	-1.5	1.0	19	561
32	1	22	6.5	-1.5	0	1.0	18	540
33	0	34	6.5	-5.5	-0.5	1.0	17	522
34	1	19	6	-7.5	0	1.0	16	450
35	0	25	6.75	-7	0	1.0	13	490
36	1	20	6.75	-3.5	0	1.0	21	569
37	0	25	6.5	-13.5	0	1.0	16	439
38	0	29	6.75	-2	-0.25	1.0	15	528
39	1	19	7	-3	-2	0.9	18	578
40	0	23	5	-4.25	-0.5	1.0	19	535
41	1	20	7.25	-4.5	-1	1.0	18	555
42	0	22	7.5	-6.25	-0.5	1.0	16	530
43	1	23	7.25	-6.25	-1.25	1.0	18	503
44	1	33	4	-3.5	-1	1.2	19	551
45	0	30	6	-2	-0.25	1.0	15	509
46	0	26	7	-8.25	-2.75	1.0	18	533
47	0	28	7	-4	-0.5	1.0	16	547
48	0	25	7	-4.75	0	1.0	15	495
49	0	21	6	-12	0	0.9	20	606

50	1	32	6.75	-4.5	-0.25	1.0	21	570
51	0	25	5.75	-5.25	-1	1.0	18	513
52	0	24	6.75	-5.5	-0.5	1.0	18	534
53	1	22	5	-5.25	-1	1.2	20	536
54	0	30	6.5	-4	-0.75	1.2	16	538
55	0	27	6.75	-2.75	-1	1.0	18	529
56	0	31	6	-6.25	-1.25	1.0	19	551
57	0	23	5.25	-5	-1.25	1.0	18	546
58	1	25	5.75	-5.5	-0.5	1.2	19	569
59	0	28	6	-2.75	-0.5	1.2	16	525
60	0	27	7.25	-3.75	0	1.2	14	509
61	0	28	7	-6	-0.75	1.0	17	549
62	1	24	6	-4.5	-2	1.0	15	537
63	1	38	5	-4	-0.25	1.0	19	555
64	0	37	6.25	-5.5	-0.25	1.0	14	529
65	0	28	7	-5.5	-1	1.0	18	518
66	0	23	7	-3.75	-1	1.0	19	601
67	0	26	7	-7.5	-0.75	1.0	21	596
68	0	26	5.5	-3.25	-0.7	1.0	15	516
69	0	26	6	-5.75	-1	1.0	20	552
70	0	27	5	-2.75	-0.5	1.0	15	538
71	0	25	6.5	-3.5	-0.25	1.0	17	576
72	1	25	6.75	-9.75	-2	1.0	16	514
73	1	30	6.75	-3.5	-0.5	1.0	18	570
74	1	22	6.75	-5.5	-0.5	1.0	17	499
75	1	23	7.25	-5.25	-0.25	1.0	15	510
76	1	30	5.5	-2.25	-0.25	1.0	18	556
77	0	20	7.25	-4.5	-0.75	1.0	20	547
78	1	18	6.5	-3.5	-1	1.0	21	580
79	0	30	6.75	-4.75	-0.25	1.0	18	560
80	1	20	6.75	-5.25	-0.25	1.0	21	556
81	1	39	5.75	-6.5	-0.25	1.0	20	544
82	1	18	6.5	-2.75	-0.75	1.0	18	551
83	0	27	6.5	-7.5	0	0.9	17	484
84	1	21	5.75	-3.25	0	1.0	15	519
85	1	24	5.75	-6.75	-0.75	1.2	19	542
86	0	26	6	-6	-0.75	1.2	15	534
87	0	23	5.7	-3.25	-0.25	1.0	18	536
88	1	22	6.25	-5.25	-0.75	1.2	16	540
89	0	22	5.75	-6.75	-0.75	1.0	16	529
90	0	23	5.75	-8	-1.5	1.0	18	506
91	0	22	6.5	-3	-0.5	1.0	15	555
92	1	22	7	-4.5	-0.5	1.2	17	515
93	0	24	7	-4.25	-0.5	1.0	21	513
94	0	24	7.25	-3.25	-1.5	1.0	15	534
95	0	32	6.5	-2	-1	1.0	17	508
96	0	30	6	-4.75	-0.25	0.9	16	569
97	0	34	6.5	-5.5	0	1.0	21	554

98	0	36	6	−3.75	−0.25	1.0	17	509
99	1	23	6.75	−7.5	−3.5	1.0	21	588
100	0	22	5.5	−4	−1	1.0	13	501
101	0	25	6.25	−8	−1.5	1.0	18	567
102	1	25	7.25	−2.75	−0.25	1.0	17	524
103	1	28	4.5	−8.5	0	1.0	15	558
104	0	29	7	−3.75	−1	1.0	16	533
105	0	31	5.5	−5.75	−1	1.0	15	489
106	0	27	5	−5.5	−0.25	1.2	16	502
107	0	32	5	−4	−1	1.0	15	527
108	0	29	3.75	−5.5	−0.75	1.0	15	516
109	0	27	4.5	−3.5	−0.25	1.0	17	520
110	0	29	4.75	−3.5	−0.25	1.0	15	545
111	1	26	6.5	−5.25	−0.75	1.0	16	510
112	0	23	5.25	−4	−0.75	1.0	15	522
113	0	29	6.75	−3.75	−0.50	1.2	20	563
114	1	22	5.5	−3.25	−0.75	1.0	16	551
115	0	25	5.75	−7.25	−0.5	1.0	17	543
116	0	23	6	−2.25	−0.25	1.2	15	545
117	0	29	6.5	−5.5	−0.5	1.2	13	523
118	1	33	6.25	−2.25	−0.25	1.2	21	605
119	0	25	5.75	−3.75	−1.25	1.0	19	562
120	0	28	7	−3.75	−0.75	1.2	16	503
121	0	26	6.75	−3.75	−0.25	1.0	15	538
122	0	26	7.75	−5.5	−0.25	1.0	16	517
123	0	25	5.25	−5.25	−1	0.9	19	569
124	0	25	6.5	−4.75	−0.5	1.0	17	517
125	0	26	6.5	−3.25	−0.25	1.0	17	518
126	0	26	5	−5	−0.75	1.0	15	515
127	0	26	7.75	−5.25	−0.5	1.0	16	514
128	0	24	7	−4.5	0	1.0	20	598
129	0	30	7.5	−3.75	0	1.2	20	573
130	0	28	6.75	−1.75	−0.5	1.0	18	569
131	0	23	6.25	−4.5	−0.25	0.9	17	528

K1(D)	K2(D)	Km(D)	x(mm)	y(mm)	Chord μ (mm)	procedure(1=SMILE, 2=FS-LASIK, 3=ICL)	post-op UDVA	post-op CDVA
43.5	44.7	44.1	0.16	-0.02	0.161245155	1	1.2	1.2
42	43	42.5	-0.01	-0.03	0.031622777	2	1.0	1.2
40.2	41.1	40.65	0.25	0.06	0.257099203	1	1.5	1.5
42.9	43.4	43.15	0.27	0.09	0.284604989	2	1.2	1.2
44.7	45.4	45.05	-0.28	-0.08	0.291204396	3	1.2	1.2
43.2	44.1	43.65	-0.08	0.09	0.120415946	2	1.0	1.2
42.7	43.8	43.25	-0.09	0.06	0.108166538	1	1.0	1.2
42.4	42.8	42.6	-0.15	0.02	0.15132746	2	1.2	1.5
43.5	45.5	44.5	-0.23	0.38	0.444184646	3	1.0	1.5
43.7	44.2	43.95	0.01	-0.11	0.11045361	2	1.5	1.5
45.6	46.3	45.95	-0.11	-0.16	0.194164878	3	1.0	1.2
45.6	46.4	46	-0.15	-0.08	0.17	2	1.5	1.5
43.4	45.8	44.6	0.02	0.11	0.111803399	3	1.2	1.2
40.3	41.7	41	-0.15	0.29	0.326496554	2	1.2	1.2
42.0	43.4	42.7	0.24	-0.10	0.26	3	1.2	1.2
42.1	43.4	42.75	0.08	0.13	0.152643375	3	1.5	1.2
43.3	44.5	43.9	-0.15	-0.09	0.174928557	3	1.2	1.2
45.1	45.9	45.5	-0.12	-0.10	0.156204994	2	1.2	1.2
45.3	47.2	46.25	-0.08	0.12	0.144222051	2	1.2	1.2
43.3	44.3	43.8	0.18	-0.17	0.247588368	2	1.2	1.2
42.7	43.7	43.2	-0.01	0.16	0.160312195	3	1.0	1.2
43.9	44.4	44.15	0	0.04	0.04	2	1.2	1.2
41.7	42.2	41.95	0.2	0.11	0.228254244	2	1.0	1.0
43.4	43.8	43.6	-0.01	0.06	0.060827625	1	1.2	1.2
43.3	44.7	44	0.01	0.22	0.220227155	3	1.2	1.2
44.1	45.2	44.65	-0.02	0.04	0.04472136	2	1.2	1.2
42.1	43.1	42.6	0.04	0.08	0.089442719	1	1.5	1.5
40.7	41.3	41	0.03	0	0.03	1	1.2	1.2
43.4	45.1	44.25	-0.02	0.1	0.10198039	2	1.0	1.0
43.3	44.8	44.05	0.09	0.1	0.13453624	2	1.2	1.2
40.5	42.2	41.35	0.37	-0.22	0.430464865	1	1.5	1.5
42.3	43.0	42.65	-0.03	-0.09	0.09486833	1	1.5	1.5
44.1	46.2	45.15	-0.03	0.05	0.058309519	2	1.2	1.2
44.3	45.9	45.1	-0.32	-0.04	0.32249031	3	1.2	1.2
44.3	45.3	44.8	-0.33	0.04	0.332415403	3	1.2	1.2
41.8	42.8	42.3	-0.07	0.29	0.298328678	2	1.0	1.0
43	44.2	43.6	0.4	0.32	0.512249939	3	1.2	1.2
42.7	43.7	43.2	0.05	0.06	0.078102497	2	1.2	1.2
39.8	42.1	40.95	0.02	0.2	0.200997512	2	1.2	1.5
44	45.2	44.6	0	0.28	0.28	1	1.2	1.2
42.8	44.3	43.55	-0.17	0.15	0.226715681	1	1.0	1.2
42.4	43.3	42.85	-0.12	0.11	0.162788206	2	1.2	1.2
43.1	44.6	43.85	0.34	-0.02	0.340587727	3	1.2	1.2
43	44.5	43.75	-0.07	0.01	0.070710678	1	1.0	1.2
41.2	42.4	41.8	-0.12	0.06	0.134164079	1	1.0	1.5
42.7	45.4	44.05	-0.16	0.1	0.188679623	3	1.2	1.2
41.9	42.6	42.25	-0.06	0.18	0.18973666	2	1.0	1.0
42.3	42.8	42.55	0.1	-0.01	0.100498756	2	1.0	1.2
45.5	46.9	46.2	0.13	0	0.13	3	1.0	1.0

42.8	43.7	43.25	0.26	-0.07	0.26925824	1	1.5	1.5
43.4	44.2	43.8	0.04	0.01	0.041231056	2	1.0	1.2
45.3	46.4	45.85	0.06	0.12	0.134164079	1	1.5	1.5
43	44.5	43.75	-0.02	-0.04	0.04472136	1	1.5	1.5
44	45.2	44.6	-0.01	0.1	0.100498756	1	1.2	1.2
43.8	45.4	44.6	-0.03	0	0.03	2	1.2	1.5
42.2	43.4	42.8	0.09	0.06	0.108166538	2	1.5	1.5
40.4	42.1	41.25	0.12	0.21	0.241867732	2	1.5	1.5
39.7	40.8	40.25	0.18	-0.02	0.181107703	1	1.0	1.2
43.3	44.1	43.7	0.12	0	0.12	2	1.5	1.5
42.2	42.6	42.4	0.05	-0.01	0.050990195	1	1.2	1.2
42.6	44.6	43.6	0.02	-0.23	0.230867928	2	1.2	1.5
40.8	42.6	41.7	-0.13	0.22	0.255538647	2	1.0	1.5
42.6	42.8	42.7	0.04	-0.01	0.041231056	2	1.5	1.5
41.8	42.0	41.9	-0.24	-0.17	0.294108823	2	1.2	1.2
41.2	42.4	41.8	0.07	0.07	0.098994949	2	1.2	1.2
41.6	42.8	42.2	0.14	0.16	0.212602916	1	1.2	1.2
44	45.2	44.6	0.27	0.36	0.45	2	1.0	1.2
40.5	41.7	41.1	0.01	0.11	0.11045361	2	1.5	1.5
42.3	43.6	42.95	-0.06	-0.05	0.078102497	1	1.2	1.2
41.4	42.1	41.75	0	0.21	0.21	2	1.0	1.2
42.4	43.4	42.9	0.12	0.09	0.15	1	1.2	1.2
41.8	43.9	42.85	0.17	-0.05	0.177200451	3	0.9	1.0
42.2	42.9	42.55	0.02	0.17	0.171172428	2	1.2	1.5
45.3	45.9	45.6	-0.28	0.12	0.304630924	2	1.2	1.2
42.1	42.4	42.25	0.08	0	0.08	2	1.2	1.2
43	43.1	43.05	-0.01	0.02	0.02236068	2	1.2	1.5
43.7	45.3	44.5	0.4	0.12	0.41761226	2	1.2	1.2
42.3	43.2	42.75	0.14	-0.07	0.156524758	1	1.2	1.2
43.6	44.5	44.05	0.04	-0.04	0.056568542	1	0.9	0.9
42.2	43.2	42.7	-0.26	-0.14	0.295296461	2	1.0	1.2
41.8	43.2	42.5	0.11	0.12	0.162788206	2	1.2	1.2
43.1	44.1	43.6	0.15	-0.19	0.242074369	1	1.0	1.0
41.9	43	42.45	-0.14	-0.18	0.228035085	3	1.2	1.5
42.3	42.6	42.45	-0.23	0.06	0.237697286	1	1.2	1.2
41.4	42.7	42.05	0.19	0.26	0.322024844	2	1.2	1.2
42.3	43.2	42.75	-0.2	-0.08	0.215406592	2	1.2	1.2
41.6	42.4	42	-0.09	0.19	0.21023796	2	1.2	1.2
40.8	42	41.4	-0.2	0.19	0.275862284	2	1.5	1.5
41.9	43	42.45	-0.02	0.13	0.131529464	2	1.0	1.0
41.8	43.4	42.6	0.28	0.05	0.284429253	3	1.0	1.0
43.4	44.5	43.95	0.02	0.32	0.320624391	1	1.0	1.2
42.3	43.1	42.7	0.01	0.04	0.041231056	2	1.2	1.2
46.2	47.7	46.95	0	0.13	0.13	2	1.2	1.2
42.2	44.5	43.35	-0.09	0.14	0.16643317	2	0.9	1.0
43.1	45.1	44.1	-0.02	0.04	0.04472136	2	1.2	1.5
43.5	44.3	43.9	-0.05	0.23	0.235372046	1	1.0	1.0
43.3	44.4	43.85	-0.1	0.23	0.250798724	1	1.2	1.2

43.2	44.1	43.65	0.05	0.04	0.064031242	2	0.9	1.2
40.2	43.5	41.85	-0.46	0.18	0.493963561	2	1.2	1.2
43.9	45.5	44.7	-0.09	0	0.09	3	1.2	1.2
44.3	46	45.15	-0.18	0.04	0.184390889	3	1.2	1.2
43.7	44.6	44.15	-0.04	-0.04	0.056568542	2	1.2	1.2
43.1	44.1	43.6	0.18	0.11	0.210950231	3	1.2	1.2
44	45.9	44.95	-0.05	0.13	0.139283883	1	1.0	1.0
45	46	45.5	0.07	0.03	0.076157731	3	1.2	1.2
43.6	44.7	44.15	0.06	0.09	0.108166538	2	1.0	1.0
44.8	47	45.9	0.28	0.11	0.300832179	2	1.2	1.2
44.1	45.4	44.75	0.04	0.09	0.098488578	2	1.2	1.5
43.9	45.5	44.7	-0.02	0.19	0.191049732	2	1.2	1.2
43.1	44.1	43.6	-0.22	0.1	0.241660919	1	1.0	1.0
43.9	45.1	44.5	0.01	0	0.01	2	1.0	1.0
43.2	44.1	43.65	-0.45	0.14	0.471274867	2	1.5	1.5
43.6	44.2	43.9	-0.04	0.25	0.253179778	2	1.2	1.2
42.8	44.5	43.65	-0.11	0.12	0.162788206	1	1.2	1.2
41.7	42.6	42.15	-0.11	0.08	0.136014705	3	1.5	1.5
42.6	43.9	43.25	0.02	-0.21	0.210950231	1	1.5	1.5
43.3	44.5	43.9	0	-0.04	0.04	2	1.5	1.5
41.8	42.6	42.2	0.02	-0.01	0.02236068	1	1.2	1.2
42.5	44.4	43.45	-0.03	0.17	0.172626765	2	1.2	1.2
43.4	44.1	43.75	0.04	0.14	0.145602198	2	1.0	1.2
43.7	44.6	44.15	-0.16	0.09	0.183575598	1	1.5	1.5
40.7	41.2	40.95	-0.11	0.05	0.12083046	2	1.0	1.0
45.4	45.7	45.55	-0.06	0.04	0.072111026	2	1.0	1.0
44.6	45.3	44.95	-0.43	-0.03	0.431045241	2	1.2	1.2
43.7	44.3	44	-0.13	-0.01	0.130384048	2	1.0	1.0
43.5	44.4	43.95	-0.01	0.16	0.160312195	2	1.2	1.2
42.2	42.8	42.5	0.12	-0.11	0.162788206	2	1.5	1.5
41.8	43.3	42.55	-0.09	-0.09	0.127279221	2	1.0	1.0
43	44.1	43.55	0.11	0.25	0.273130006	2	1.2	1.2
42.4	42.8	42.6	-0.08	0.17	0.187882942	2	1.2	1.2
43	43.9	43.45	-0.28	0.16	0.32249031	1	1.2	1.2

post-op sphere (D)	post-op cylinder (D)	glare	galre-frequency	glare-severity	glareborthersome	halos	halos-frequency	halos-severity	halos-borthersome
-0.25	0	2	1	1	0	0	0	0	0
-0.25	0	0	0	0	0	0	0	0	0
0	0	2	1	0	1	0	0	0	0
0	0	1	1	0	0	1	1	0	0
0	0	0	0	0	0	3	1	1	1
0	-0.25	2	1	1	0	2	1	1	0
0	-0.5	0	0	0	0	0	0	0	0
-0.25	0	4	2	1	1	3	1	1	1
-0.25	-0.75	3	1	1	1	4	2	1	1
0.5	-0.25	0	0	0	0	0	0	0	0
0	0	0	0	0	0	3	3	0	0
0.5	0	2	1	1	0	0	0	0	0
0	-0.5	3	1	1	1	6	3	2	1
0	0	3	1	1	1	3	1	1	1
0	0	2	1	1	0	5	2	2	1
0	0	0	0	0	0	2	1	1	0
0	0	0	0	0	0	0	0	0	0
-0.25	0	2	1	1	0	3	1	1	1
0.25	0	1	1	0	0	0	0	0	0
0	0	2	1	1	0	0	0	0	0
-0.25	0	0	0	0	0	5	2	2	1
0	0	3	1	1	1	0	0	0	0
0	0	3	1	1	1	5	2	2	1
0	0	5	1	2	2	4	1	1	2
0	0	5	3	1	1	4	3	1	0
-0.25	0	0	0	0	0	0	0	0	0
0	0	2	1	1	0	0	0	0	0
-0.25	0	3	1	1	1	3	1	1	1
-0.25	-0.5	3	1	1	1	0	0	0	0
-0.25	0	3	1	1	1	4	2	1	1
-0.25	-0.5	5	1	3	1	3	1	1	1
0	0	3	1	1	1	3	1	1	1
-0.25	0	4	1	2	1	3	1	1	1
0.25	-1	4	3	1	0	0	0	0	0
0	0	4	1	2	1	3	1	1	1
-0.25	-0.5	3	1	1	1	2	1	1	0
0	0	3	1	1	1	2	1	1	0
0.25	0	3	1	1	1	3	1	1	1
0	-0.5	3	1	1	1	0	0	0	0
0	0	2	1	1	0	0	0	0	0
-0.25	0	3	1	1	1	0	0	0	0
-0.25	0	0	0	0	0	1	1	0	0
-0.5	-0.5	2	1	1	0	2	1	1	0
0.25	-0.75	2	1	1	0	3	1	1	1
0.5	-0.25	0	0	0	0	0	0	0	0
0	0	6	3	2	1	6	3	2	1
0	0	3	1	1	1	3	1	1	1
-0.5	0	1	1	0	0	0	0	0	0
0	0	4	2	1	1	5	3	1	1

0	0	0	0	0	0	0	0	0	0
0.25	-0.25	0	0	0	0	1	1	0	0
0	0	3	1	1	1	3	1	1	1
-0.25	0	0	0	0	0	0	0	0	0
0.5	0	3	1	1	1	0	0	0	0
0.5	-0.5	3	1	1	1	3	1	1	1
0	0	0	0	0	0	1	1	0	0
0.25	0	2	1	1	0	0	0	0	0
-0.25	0	3	1	1	1	0	0	0	0
0	0	3	1	1	1	0	0	0	0
0.25	0	4	1	2	1	0	0	0	0
0.5	-0.25	3	1	1	1	0	0	0	0
1	-0.75	4	2	1	1	5	2	2	1
0	0	0	0	0	0	0	0	0	0
0	-0.5	6	3	2	1	4	2	1	1
0	0	2	1	1	0	2	1	1	0
0	0	2	1	1	0	0	0	0	0
-0.75	-0.5	2	1	1	0	0	0	0	0
0	0	5	3	1	1	3	1	1	1
0.25	-0.25	3	1	1	1	0	0	0	0
-0.25	0	3	1	1	1	3	1	1	1
0	0	0	0	0	0	2	1	1	0
-0.5	0	6	3	2	1	6	3	2	1
0	-0.5	3	1	1	1	0	0	0	0
0	0	3	1	1	1	0	0	0	0
0	0	3	1	1	1	2	1	1	0
-0.5	0	3	1	1	1	6	3	2	1
0	0	0	0	0	0	3	2	1	0
0.25	-0.25	3	1	1	1	0	0	0	0
0	0	3	1	1	1	4	2	1	1
-0.25	-0.25	3	1	1	1	0	0	0	0
0.25	-0.5	6	3	2	1	0	0	0	0
0	0	2	1	1	0	0	0	0	0
0	-0.5	0	0	0	0	3	1	1	1
0	0	0	0	0	0	2	1	1	0
0	0	0	0	0	0	3	1	1	1
0.25	0	3	1	1	1	0	0	0	0
0	0	1	1	0	0	1	1	0	0
0	0	2	1	1	0	2	1	1	0
-0.25	0	5	2	2	1	4	1	2	1
0	0	4	2	1	1	5	2	2	1
0.5	0	3	1	1	1	3	1	1	1
0	0	7	3	2	2	0	0	0	0
0	0	3	1	1	1	2	1	1	0
0	-0.5	3	1	1	1	3	1	1	1
0	0	3	1	1	1	3	1	1	1
0	0	5	2	2	1	5	3	1	1
0	0	3	1	1	1	3	1	1	1

-0.5	0	3	1	1	1	3	1	1	1
0	0	4	2	1	1	4	2	1	1
0	0	6	3	2	1	4	2	1	1
0	0	3	1	1	1	4	2	1	1
0	0	3	1	1	1	0	0	0	0
0	0	3	1	1	1	2	1	1	0
0	0	3	1	1	1	2	1	1	0
0	0	3	1	1	1	0	0	0	0
0.25	0	0	0	0	0	1	1	0	0
-0.25	0	0	0	0	0	2	1	1	0
0.25	0	3	1	1	1	0	0	0	0
0	-0.25	3	1	1	1	2	1	1	0
0	0	0	0	0	0	0	0	0	0
0	0	0	0	0	0	2	1	1	0
0	0	1	1	0	0	4	2	1	1
0	0	2	1	1	0	0	0	0	0
0	0	0	0	0	0	0	0	0	0
0	0	5	2	2	1	5	2	2	1
0	0	3	1	1	1	0	0	0	0
0	0	0	0	0	0	0	0	0	0
0	0	5	2	2	1	6	3	2	1
0	0	0	0	0	0	2	1	1	0
0	-0.5	5	2	1	2	0	0	0	0
0	0	0	0	0	0	0	0	0	0
0	0	8	3	3	2	3	1	1	1
0	0	0	0	0	0	0	0	0	0
0	0	3	1	1	1	3	1	1	1
0	0	8	3	3	2	3	1	1	1
0	0	0	0	0	0	0	0	0	0
0	0	1	1	0	0	1	1	0	0
0	-0.5	3	1	1	1	0	0	0	0
0	0	3	1	1	1	3	1	1	1
0	0	0	0	0	0	0	0	0	0
0	0	0	0	0	0	2	1	0	1

starburst	starburst-frequency	starburst-severity	starburst-borthersome	hazy vision	hazy vision-frequency	hazy vision-severity
0	0	0	0	3	1	1
0	0	0	0	5	2	2
2	1	0	1	3	1	1
2	1	0	1	0	0	0
0	0	0	0	0	0	0
0	0	0	0	2	1	1
0	0	0	0	0	0	0
3	1	1	1	3	1	1
3	1	1	1	0	0	0
0	0	0	0	0	0	0
0	0	0	0	0	0	0
2	1	1	0	0	0	0
0	0	0	0	1	1	0
3	2	1	0	3	1	1
3	1	1	1	1	1	0
0	0	0	0	0	0	0
0	0	0	0	0	0	0
0	0	0	0	3	1	1
2	1	1	0	0	0	0
0	0	0	0	0	0	0
0	0	0	0	3	1	1
0	0	0	0	0	0	0
0	0	0	0	3	1	1
4	0	2	2	9	3	3
0	0	0	0	0	0	0
6	3	2	1	3	1	1
0	0	0	0	3	1	1
0	0	0	0	0	0	0
0	0	0	0	0	0	0
0	0	0	0	0	0	0
0	0	0	0	3	1	1
0	0	0	0	0	0	0
0	0	0	0	0	0	0
0	0	0	0	0	0	0
5	2	2	1	1	1	0
0	0	0	0	3	1	1
0	0	0	0	2	1	1
0	0	0	0	4	1	2
4	2	1	1	0	0	0
0	0	0	0	0	0	0
0	0	0	0	0	0	0
0	0	0	0	0	0	0
2	1	1	0	0	0	0
0	0	0	0	3	1	1
5	2	2	1	0	0	0
0	0	0	0	1	1	0
0	0	0	0	0	0	0
2	1	1	0	0	0	0
4	2	1	1	5	1	2

0	0	0	0	2	1	1
0	0	0	0	0	0	0
2	1	1	0	2	1	1
2	1	1	0	0	0	0
0	0	0	0	0	0	0
0	0	0	0	3	1	1
0	0	0	0	0	0	0
0	0	0	0	0	0	0
3	1	1	1	0	0	0
0	0	0	0	3	1	1
3	1	1	1	0	0	0
0	0	0	0	3	1	1
6	3	1	2	3	1	1
3	1	1	1	0	0	0
2	1	1	0	4	2	1
0	0	0	0	2	1	1
0	0	0	0	0	0	0
2	1	1	0	3	1	1
5	3	1	1	4	2	1
2	1	1	0	3	1	1
3	1	1	1	5	3	1
2	1	1	0	0	0	0
0	0	0	0	2	1	1
0	0	0	0	0	0	0
0	0	0	0	3	1	1
2	1	1	0	0	0	0
0	0	0	0	0	0	0
0	0	0	0	0	0	0
0	0	0	0	0	0	0
0	0	0	0	3	1	1
0	0	0	0	0	0	0
0	0	0	0	0	0	0
2	1	1	0	0	0	0
0	0	0	0	0	0	0
0	0	0	0	0	0	0
2	1	1	0	0	0	0
2	1	1	0	0	0	0
0	0	0	0	1	1	0
0	0	0	0	3	1	1
2	1	1	0	1	1	0
3	1	1	1	3	1	1
0	0	0	0	0	0	0
0	0	0	0	0	0	0
2	1	1	0	0	0	0
4	2	1	1	0	0	0
0	0	0	0	3	1	1
2	1	1	0	3	1	1
0	0	0	0	3	1	1

3	1	1	1	3	1	1
0	0	0	0	1	1	0
1	1	0	0	0	0	0
0	0	0	0	3	1	1
0	0	0	0	0	0	0
0	0	0	0	2	1	1
0	0	0	0	3	1	1
0	0	0	0	0	0	0
0	0	0	0	0	0	0
3	1	1	1	0	0	0
3	1	1	1	0	0	0
3	1	1	1	0	0	0
0	0	0	0	0	0	0
0	0	0	0	0	0	0
0	0	0	0	3	1	1
2	1	1	0	3	1	1
3	1	1	1	0	0	0
0	0	0	0	2	1	1
1	1	0	0	3	1	1
0	0	0	0	0	0	0
4	1	1	2	4	1	2
3	1	1	1	0	0	0
4	1	1	2	3	1	1
0	0	0	0	0	0	0
8	3	3	2	3	1	1
0	0	0	0	3	1	1
3	1	1	1	0	0	0
5	2	2	1	3	1	1
0	0	0	0	0	0	0
0	0	0	0	1	1	0
0	0	0	0	3	1	1
3	1	1	1	0	0	0
0	0	0	0	0	0	0
0	0	0	0	3	1	1

borthersome	blurred vision	blurred vision-frequency	blurred vision-severity	blurred vision-borthersome	distortion	distortion-frequency	distortion-severity
1	0	0	0	0	0	0	0
1	3	1	1	1	0	0	0
1	0	0	0	0	0	0	0
0	2	1	0	1	0	0	0
0	0	0	0	0	0	0	0
0	2	1	1	0	0	0	0
0	3	1	1	1	0	0	0
1	3	1	1	1	0	0	0
0	1	1	0	0	0	0	0
0	0	0	0	0	0	0	0
0	0	0	0	0	0	0	0
0	0	0	0	0	0	0	0
0	0	0	0	0	0	0	0
1	0	0	0	0	0	0	0
0	0	0	0	0	0	0	0
0	0	0	0	0	0	0	0
0	0	0	0	0	0	0	0
1	3	1	1	1	0	0	0
0	0	0	0	0	0	0	0
0	0	0	0	0	0	0	0
1	3	1	1	1	0	0	0
0	0	0	0	0	0	0	0
1	0	0	0	0	0	0	0
3	8	3	2	3	0	0	0
0	0	0	0	0	0	0	0
1	3	1	1	1	0	0	0
1	2	1	1	0	0	0	0
0	1	1	0	0	0	0	0
0	3	1	1	1	0	0	0
0	3	1	1	1	0	0	0
1	3	1	1	1	0	0	0
0	0	0	0	0	0	0	0
0	0	0	0	0	0	0	0
0	0	0	0	0	0	0	0
0	2	1	0	1	0	0	0
1	3	1	1	1	0	0	0
0	1	1	0	0	0	0	0
1	0	0	0	0	0	0	0
0	1	1	0	0	0	0	0
0	0	0	0	0	0	0	0
0	0	0	0	0	0	0	0
0	0	0	0	0	0	0	0
0	0	0	0	0	0	0	0
1	3	1	1	1	0	0	0
0	0	0	0	0	0	0	0
0	1	1	0	0	0	0	0
0	3	1	1	1	0	0	0
0	0	0	0	0	0	0	0
2	3	1	1	1	0	0	0

0	0	0	0	0	0	0	0
0	2	1	1	0	0	0	0
0	0	0	0	0	0	0	0
0	5	2	2	1	0	0	0
0	3	1	1	1	0	0	0
1	3	1	1	1	0	0	0
0	0	0	0	0	0	0	0
0	0	0	0	0	0	0	0
0	1	1	0	0	0	0	0
1	0	0	0	0	0	0	0
0	0	0	0	0	0	0	0
1	3	1	1	1	0	0	0
1	8	3	2	3	0	0	0
0	2	1	1	0	0	0	0
1	4	1	2	1	0	0	0
0	0	0	0	0	0	0	0
0	2	1	1	0	0	0	0
1	3	1	1	1	0	0	0
1	3	1	1	1	0	0	0
1	2	1	1	0	2	1	1
1	5	3	1	1	3	1	1
0	0	0	0	0	0	0	0
0	0	0	0	0	0	0	0
0	0	0	0	0	0	0	0
1	3	1	1	1	0	0	0
0	0	0	0	0	0	0	0
0	0	0	0	0	0	0	0
0	0	0	0	0	0	0	0
0	0	0	0	0	0	0	0
1	4	2	1	1	0	0	0
0	0	0	0	0	0	0	0
0	5	1	3	1	0	0	0
0	0	0	0	0	0	0	0
0	2	1	0	1	0	0	0
0	2	1	1	0	0	0	0
0	0	0	0	0	0	0	0
0	3	1	1	1	0	0	0
0	0	0	0	0	0	0	0
1	4	2	1	1	0	0	0
0	3	1	1	1	0	0	0
1	3	1	1	1	0	0	0
0	3	1	1	1	0	0	0
0	4	2	1	1	0	0	0
0	0	0	0	0	0	0	0
0	5	2	2	1	0	0	0
1	3	1	1	1	0	0	0
1	3	1	1	1	0	0	0
1	3	1	1	1	0	0	0

1	3	1	1	1	0	0	0
0	0	0	0	0	0	0	0
0	0	0	0	0	0	0	0
1	3	1	1	1	0	0	0
0	0	0	0	0	0	0	0
0	3	1	1	1	0	0	0
1	3	1	1	1	0	0	0
0	0	0	0	0	0	0	0
0	3	1	1	1	0	0	0
0	0	0	0	0	0	0	0
0	0	0	0	0	0	0	0
0	3	1	1	1	0	0	0
0	0	0	0	0	0	0	0
0	3	1	1	1	0	0	0
1	3	1	1	1	0	0	0
1	2	1	1	0	0	0	0
0	2	1	1	0	0	0	0
0	2	1	1	0	0	0	0
1	1	1	0	0	0	0	0
0	0	0	0	0	0	0	0
1	4	1	1	2	0	0	0
0	0	0	0	0	0	0	0
1	0	0	0	0	0	0	0
0	2	1	1	0	0	0	0
1	4	1	2	1	0	0	0
1	3	1	1	1	0	0	0
0	2	1	1	0	2	1	1
1	3	1	1	1	0	0	0
0	0	0	0	0	0	0	0
0	0	0	0	0	0	0	0
1	4	2	1	1	0	0	0
0	3	1	1	1	0	0	0
0	3	1	1	1	0	0	0
1	3	2	0	1	0	0	0

distortion-borthersome	double images	double images-frequency	double images-severity	double images-borthersome	fluctuation in vision
0	0	0	0	0	2
0	3	1	1	1	3
0	0	0	0	0	3
0	0	0	0	0	0
0	4	1	1	2	5
0	0	0	0	0	2
0	0	0	0	0	3
0	4	2	1	1	2
0	0	0	0	0	2
0	0	0	0	0	0
0	0	0	0	0	1
0	0	0	0	0	0
0	0	0	0	0	1
0	0	0	0	0	3
0	0	0	0	0	2
0	0	0	0	0	0
0	0	0	0	0	0
0	0	0	0	0	3
0	0	0	0	0	3
0	0	0	0	0	0
0	3	1	1	1	3
0	0	0	0	0	3
0	0	0	0	0	2
0	0	0	0	0	8
0	0	0	0	0	0
0	3	1	1	1	3
0	3	1	1	1	3
0	0	0	0	0	3
0	3	1	1	1	3
0	0	0	0	0	1
0	3	1	1	1	0
0	0	0	0	0	3
0	0	0	0	0	3
0	0	0	0	0	0
0	0	0	0	0	0
0	0	0	0	0	3
0	1	1	0	0	1
0	4	1	2	1	4
0	0	0	0	0	0
0	0	0	0	0	0
0	0	0	0	0	2
0	0	0	0	0	0
0	0	0	0	0	3
0	0	0	0	0	3
0	0	0	0	0	0
0	3	1	1	1	1
0	0	0	0	0	3
0	0	0	0	0	4
0	3	1	1	1	3

0	0	0	0	0	2
0	0	0	0	0	2
0	0	0	0	0	0
0	0	0	0	0	2
0	0	0	0	0	0
0	3	1	1	1	3
0	0	0	0	0	2
0	0	0	0	0	1
0	0	0	0	0	0
0	3	1	1	1	3
0	0	0	0	0	2
0	3	1	1	1	3
0	3	1	1	1	3
0	0	0	0	0	4
0	0	0	0	0	0
0	0	0	0	0	2
0	0	0	0	0	0
0	0	0	0	0	3
0	0	0	0	0	4
0	1	1	0	0	1
1	3	1	1	1	3
0	3	1	1	1	3
0	0	0	0	0	3
0	0	0	0	0	3
0	0	0	0	0	3
0	0	0	0	0	1
0	0	0	0	0	3
0	0	0	0	0	2
0	0	0	0	0	0
0	0	0	0	0	3
0	0	0	0	0	2
0	0	0	0	0	5
0	0	0	0	0	3
0	0	0	0	0	1
0	2	1	1	0	0
0	0	0	0	0	3
0	0	0	0	0	3
0	0	0	0	0	0
0	0	0	0	0	3
0	1	1	0	0	0
0	3	1	1	1	3
0	3	1	1	1	3
0	0	0	0	0	3
0	0	0	0	0	3
0	3	1	1	1	1
0	0	0	0	0	5
0	0	0	0	0	3
0	0	0	0	0	3

0	0	0	0	0	3
0	0	0	0	0	3
0	0	0	0	0	0
0	0	0	0	0	3
0	0	0	0	0	0
0	0	0	0	0	3
0	3	1	1	1	3
0	0	0	0	0	0
0	0	0	0	0	3
0	0	0	0	0	0
0	0	0	0	0	3
0	0	0	0	0	2
0	0	0	0	0	0
0	0	0	0	0	3
0	0	0	0	0	0
0	0	0	0	0	3
0	0	0	0	0	2
0	0	0	0	0	2
0	0	0	0	0	3
0	0	0	0	0	0
0	4	1	2	2	5
0	0	0	0	0	3
0	3	1	1	1	4
0	0	0	0	0	0
0	0	0	0	0	3
0	0	0	0	0	0
0	2	1	1	0	0
0	3	1	1	1	2
0	0	0	0	0	0
0	2	1	1	0	2
0	0	0	0	0	5
0	3	1	1	1	3
0	0	0	0	0	0
0	0	0	0	0	0

fluctuation in vision-frequency	fluctuation in vision-severity	fluctuation in vision-borthersome	focusing difficulty	focusing difficulty-frequency	focusing difficulty-severity	focusing difficulty-borthersome
1	1	0	1	1	0	0
1	1	1	7	3	2	2
1	1	1	3	1	1	1
0	0	0	0	0	0	0
2	1	2	3	1	1	1
1	1	0	0	0	0	0
1	1	1	0	0	0	0
1	1	0	3	1	1	1
1	1	0	0	0	0	0
0	0	0	0	0	0	0
1	0	0	0	0	0	0
0	0	0	0	0	0	0
1	0	0	0	0	0	0
1	1	1	0	0	0	0
1	1	0	0	0	0	0
0	0	0	0	0	0	0
0	0	0	0	0	0	0
1	1	1	3	1	1	1
1	1	1	0	0	0	0
0	0	0	0	0	0	0
1	1	1	3	1	1	1
1	1	1	0	0	0	0
1	1	0	0	0	0	0
3	2	3	7	3	2	2
0	0	0	0	0	0	0
1	1	1	0	0	0	0
1	1	1	3	1	1	1
1	1	1	0	0	0	0
1	1	1	0	0	0	0
1	0	0	0	0	0	0
0	0	0	0	0	0	0
1	1	1	3	1	1	1
1	1	1	3	1	1	1
0	0	0	0	0	0	0
0	0	0	0	0	0	0
1	1	1	1	1	0	0
1	0	0	0	0	0	0
2	1	1	3	1	1	1
0	0	0	0	0	0	0
0	0	0	0	0	0	0
1	1	0	0	0	0	0
0	0	0	0	0	0	0
1	1	1	2	1	1	0
1	1	1	3	1	1	1
0	0	0	0	0	0	0
1	0	0	0	0	0	0
1	1	1	0	0	0	0
1	2	1	3	1	1	1
1	1	1	4	1	2	1

1	1	0	0	0	0	0
1	1	0	3	1	1	1
0	0	0	0	0	0	0
1	1	0	0	0	0	0
0	0	0	3	1	1	1
1	1	1	3	1	1	1
1	1	0	3	1	1	1
1	0	0	0	0	0	0
0	0	0	1	1	0	0
1	1	1	0	0	0	0
1	1	0	1	1	0	0
1	1	1	3	1	1	1
1	1	1	9	3	3	3
2	1	1	0	0	0	0
0	0	0	0	0	0	0
1	1	0	3	1	1	1
0	0	0	1	1	0	0
1	1	1	0	0	0	0
2	1	1	3	1	1	1
1	0	0	5	2	2	1
1	1	1	3	1	1	1
1	1	1	2	1	1	0
2	1	0	2	1	1	0
1	1	1	0	0	0	0
1	1	1	0	0	0	0
1	0	0	0	0	0	0
1	1	1	3	1	1	1
1	1	0	2	1	1	0
0	0	0	0	0	0	0
1	1	1	3	1	1	1
1	1	0	0	0	0	0
1	3	1	3	1	1	1
1	1	1	3	1	1	1
1	0	0	0	0	0	0
0	0	0	1	1	0	0
1	1	1	0	0	0	0
1	1	1	3	1	1	1
0	0	0	0	0	0	0
1	1	1	6	2	2	2
0	0	0	0	0	0	0
1	1	1	3	1	1	1
1	1	1	3	1	1	1
1	1	1	0	0	0	0
1	1	1	3	1	1	1
0	0	0	3	1	1	1
2	2	1	3	1	1	1
1	1	1	3	1	1	1
1	1	1	3	1	1	1

1	1	1	7	3	2	2
1	1	1	0	0	0	0
0	0	0	2	1	1	0
1	1	1	3	1	1	1
0	0	0	0	0	0	0
1	1	1	3	1	1	1
1	1	1	3	1	1	1
0	0	0	0	0	0	0
1	1	1	1	1	0	0
0	0	0	3	1	1	1
1	1	1	3	1	1	1
1	1	0	3	1	1	1
0	0	0	0	0	0	0
1	1	1	2	1	1	0
0	0	0	0	0	0	0
1	1	1	3	1	1	1
1	1	0	3	1	1	1
1	1	0	0	0	0	0
1	1	1	4	2	1	1
0	0	0	0	0	0	0
2	1	2	6	2	2	2
1	1	1	3	1	1	1
2	1	1	0	0	0	0
0	0	0	0	0	0	0
1	1	1	3	1	1	1
0	0	0	0	0	0	0
0	0	0	0	0	0	0
1	0	1	3	1	1	1
0	0	0	0	0	0	0
1	1	0	0	0	0	0
2	1	2	6	2	2	2
1	1	1	3	1	1	1
0	0	0	0	0	0	0
0	0	0	2	1	1	0

depth perception	depth perception-frequency	depth perception-severity	depth perception-borthersom	total scores	total socres(ordinal)	total frequency scores	total severity scores
0	0	0	0	8	1	4	3
0	0	0	0	21	3	8	7
0	0	0	0	13	2	5	3
0	0	0	0	6	1	4	0
3	1	1	1	18	2	6	5
0	0	0	0	10	2	5	5
0	0	0	0	6	1	2	2
2	1	1	0	27	3	11	9
0	0	0	0	13	2	6	4
0	0	0	0	0	1	0	0
0	0	0	0	4	1	4	0
0	0	0	0	4	1	2	2
0	0	0	0	11	2	6	3
0	0	0	0	15	2	6	5
0	0	0	0	13	2	6	5
0	0	0	0	2	1	1	1
0	0	0	0	0	1	0	0
0	0	0	0	17	2	6	6
0	0	0	0	6	1	3	2
0	0	0	0	2	1	1	1
0	0	0	0	20	3	7	7
0	0	0	0	6	1	2	2
0	0	0	0	13	2	5	5
9	3	3	3	54	4	17	17
0	0	0	0	9	1	6	2
0	0	0	0	18	2	7	6
0	0	0	0	16	2	6	6
0	0	0	0	10	2	4	3
0	0	0	0	12	2	4	4
0	0	0	0	11	2	5	3
0	0	0	0	17	2	5	7
0	0	0	0	12	2	4	4
0	0	0	0	13	2	4	5
0	0	0	0	4	1	3	1
0	0	0	0	15	2	6	5
0	0	0	0	15	2	6	5
0	0	0	0	10	2	6	3
6	2	2	2	27	3	9	10
0	0	0	0	8	1	4	2
0	0	0	0	2	1	1	1
0	0	0	0	5	1	2	2
0	0	0	0	1	1	1	0
2	1	1	0	13	2	6	6
0	0	0	0	17	2	6	6
3	1	1	1	8	1	3	3
0	0	0	0	18	2	10	5
3	1	1	1	15	2	5	5
0	0	0	0	10	2	4	4
0	0	0	0	31	4	12	10

0	0	0	0	4	1	2	2
0	0	0	0	8	1	4	3
0	0	0	0	10	2	4	4
0	0	0	0	9	1	4	4
0	0	0	0	9	1	3	3
0	0	0	0	21	3	7	7
0	0	0	0	6	1	3	2
0	0	0	0	3	1	2	1
0	0	0	0	8	1	4	2
3	1	1	1	15	2	5	5
0	0	0	0	10	2	4	4
0	0	0	0	18	2	6	6
3	1	1	1	44	4	17	13
0	0	0	0	9	1	4	3
0	0	0	0	20	3	9	7
0	0	0	0	11	2	5	5
0	0	0	0	5	1	3	2
0	0	0	0	13	2	5	5
4	2	1	1	31	4	15	8
0	0	0	0	19	2	9	7
3	1	1	1	34	4	14	10
0	0	0	0	12	2	5	5
0	0	0	0	19	2	10	7
0	0	0	0	6	1	2	2
3	1	1	1	15	2	5	5
0	0	0	0	8	1	4	3
0	0	0	0	15	2	6	5
0	0	0	0	7	1	4	3
0	0	0	0	3	1	1	1
0	0	0	0	20	3	8	6
3	1	1	1	8	1	3	3
0	0	0	0	19	2	6	9
0	0	0	0	10	2	4	4
0	0	0	0	6	1	3	1
0	0	0	0	7	1	4	3
0	0	0	0	8	1	3	3
0	0	0	0	14	2	5	5
0	0	0	0	3	1	3	0
3	1	1	1	23	3	9	8
0	0	0	0	16	2	7	6
0	0	0	0	27	3	10	9
0	0	0	0	18	2	6	6
0	0	0	0	14	2	6	4
0	0	0	0	13	2	5	5
0	0	0	0	22	3	8	7
3	1	1	1	23	3	8	8
3	1	1	1	27	3	11	9
0	0	0	0	18	2	6	6

0	0	0	0	25	3	9	8
0	0	0	0	12	2	6	3
0	0	0	0	13	2	7	4
0	0	0	0	19	2	7	6
0	0	0	0	3	1	1	1
0	0	0	0	16	2	6	6
0	0	0	0	20	3	7	7
0	0	0	0	3	1	1	1
0	0	0	0	8	1	4	2
0	0	0	0	8	1	3	3
3	1	1	1	15	2	5	5
0	0	0	0	16	2	6	6
0	0	0	0	0	1	0	0
0	0	0	0	10	2	4	4
0	0	0	0	11	2	5	3
0	0	0	0	15	2	6	6
0	0	0	0	10	2	4	4
0	0	0	0	16	2	7	7
3	1	1	1	18	2	8	5
0	0	0	0	0	1	0	0
3	1	1	1	41	4	14	14
3	1	1	1	14	2	5	5
0	0	0	0	19	2	7	5
0	0	0	0	2	1	1	1
0	0	0	0	32	4	11	12
0	0	0	0	6	1	2	2
0	0	0	0	15	2	6	6
0	0	0	0	30	4	11	10
0	0	0	0	0	1	0	0
0	0	0	0	7	1	5	2
0	0	0	0	21	3	8	6
3	1	1	1	24	3	8	8
0	0	0	0	3	1	1	1
0	0	0	0	10	2	5	2

total borthersome scores
1
6
5
2
7
0
2
7
3
0
0
0
2
4
2
0
0
5
1
0
6
2
3
20
1
5
4
3
4
3
5
4
4
0
4
4
1
8
2
0
1
0
1
5
2
3
5
2
9

0
1
2
1
3
7
1
0
2
5
2
6
14
2
4
1
0
3
8
3
10
2
2
2
5
1
4
0
1
6
2
4
2
2
0
2
4
0
6
3
8
6
4
3
6
7
7
6

8
3
2
6
1
4
6
1
2
2
5
4
0
2
3
3
2
2
5
0
14
4
7
0
9
2
3
9
0
0
7
8
1
3
